# Comorbidity and the association with 1-year mortality in hip fracture patients: can the ASA score and the Charlson Comorbidity Index be used interchangeably?

**DOI:** 10.1007/s40520-021-01896-x

**Published:** 2021-06-09

**Authors:** Stina Ek, Anna C. Meyer, Margareta Hedström, Karin Modig

**Affiliations:** 1 Unit of Epidemiology, Institute of Environmental Medicine, Karolinska Institutet, Stockholm, Sweden; 2 Department of Orthopedics, Karolinska University Hospital, Stockholm, Sweden; 3 Department of Clinical Science, Intervention and Technology, Karolinska Institutet, Stockholm, Sweden

**Keywords:** Older adults, National register, Injury, Geriatric

## Abstract

**Background:**

Charlson Comorbidity Index (CCI) has been suggested to be associated with mortality in hip fracture patients, to the same extent as more expensive and time-consuming tools. However, even CCI might be too time-consuming in a clinical setting.

**Aim:**

To investigate whether the American Society of Anaesthesiologists score (ASA score), a simple grading from the anaesthesiologist’s examination, is comparable with CCI in the association with 1-year mortality after a hip fracture.

**Methods:**

The study population was patients 60 + years registered in the Swedish Hip Fracture Registry with a first-time hip fracture between 1997 and 2017 (*N* = 165,596). The outcome was 1-year mortality, and the exposures were ASA score and CCI. The association between comorbidity and mortality was described with Kaplan–Meier curves and analyzed with Cox proportional hazards models.

**Results:**

The Kaplan–Meier curves showed a stepwise increase in mortality for increasing values of both ASA and CCI. The Hazard Ratios (HRs) for the highest ASA (4–5) were **3.8** (95% Confidence Interval 3.5–4.2) for women and **3.2** (2.8–3.6) for men in the fully adjusted models. Adjusted HRs for the highest CCI (4 +) were **3.6** (3.3–3.9) for women and **2.5** (2.3–2.7) for men. Reference was the lowest score value for both tools. The correlation between the tools was moderate.

**Conclusions:**

Both ASA and CCI show a similar stepwise association with 1-year mortality in hip fracture patients, despite measuring different factors and capturing different individuals at risk. Since the ASA score is already accessible for health care staff, it might be preferable to aid in prioritizing vulnerable hip fracture patients at risk of adverse outcomes.

**Supplementary Information:**

The online version contains supplementary material available at 10.1007/s40520-021-01896-x.

## Introduction

Excess mortality following a hip fracture is a major public health concern among older adults, with a mortality rate twice as high as for the general population [1]. Hence, it is vital to understand which individuals are at a higher risk for adverse outcomes after a hip fracture, to be able to target pre-, per- and postoperative interventions.

Several clinical tools that build on comorbidities have been suggested to predict mortality after a hip fracture, among them the enUmeration of Mortality and Morbidity (O-POSSUM) and the Nottingham Hip Fracture Score (NHFS) [2, 3]. However, in addition to being expensive and time-consuming, their prediction of mortality is far from perfect [4]. These tools often rely on medical history and related comorbidities, and a high level of comorbidities at the time of the hip fracture is known to increase the risk of subsequent mortality [5–8]. The Charlson Comorbidity Index (CCI), which builds on information on specific comorbidities from previous hospital admissions, has been suggested to be a less expensive and time-consuming alternative to predict mortality among hip fracture patients [9–11]. Several studies have shown that CCI is associated with 1-year mortality after a hip fracture [6–8, 12]. However, to go back and investigate every individual patient’s medical record in an acute clinical setting might not be feasible.

The American Society of Anaesthesiologists classification of physical status (ASA score) is a clinical estimate of the patient’s overall health made by an anaesthesiologist before surgery, with a scoring ranging from 1 to 6, where an ASA score of 1 represents “healthy” and a score of 6 means that the patient is deceased [13–15]. Studies with various follow-up periods have shown an association between ASA score and mortality after hip fracture [8, 16, 17]. In particular, an ASA score of three or more has shown to be associated with higher mortality [8]. In addition, ASA score is also a good predictor of the risk of post-operative complications and re-admission, both possibly associated with an increased mortality rate [18, 19]. The most common use of ASA score in hip fracture research is to adjust for it as a proxy for health status, when exploring risk factors for other adverse outcomes such as surgery failure and reoperations, which is also closely related to increased mortality [20].

Although both tools evaluate patient’s health status and risk for excess mortality, the construct of them differs considerably. The main differences are that ASA score gives a measure of the patients’ general health at the time of surgery, while CCI mirrors a history of specific diseases. Therefore, it is of interest to study whether ASA score, a simpler measure that will be available from the anaesthesiologist’s notes from the same hospitalization, is comparable with CCI in the association with mortality after hip fracture. In fact, a recent study on 320 patients from Australia has compared these tools to predict mortality among hip fracture patients and found that ASA score, but not CCI was associated with 1 year mortality, although additional studies with larger samples are needed [21].

We hypothesize that both ASA score and CCI are associated with higher mortality in this patient group, although whether the tools capture the same individuals is unclear since the measures are constructed in different ways. It is also of interest to see if the two measures differ in how they predict mortality for men and women, respectively. In addition, whether the higher mortality among male hip fracture patients can be explained by higher comorbidity levels. The aim of this study was to investigate whether the ASA score is comparable to CCI in the association with 1-year mortality in patients with a first-time hip fracture, and whether this association differed between women and men.

## Methods

This cohort study was based on a combination of the Swedish National Hip Fracture Registry (SHR, Rikshöft), the Swedish Patient Registry (NPR), the Integrated Database for Labour market Research (LISA) and the Cause of Death Registry. The SHR is a clinical register, covering about 80% of all hip fractures in Sweden [22]. The NPR and the Cause of Death Registry are administrative registers and have a close to full national coverage [23]. All sources are linked to each other by the Swedish Personal Number (PIN) and thereafter anonymized with each individual receiving a unique id number replacing the PIN.

We included all patients registered in the SHR with a first-time hip fracture above the age of 60 years, between the years 1997 and 2017 (*N* = 194,145). After excluding pathologic fractures (*n* = 3 279), individuals that did not receive surgery (*n* = 474), individuals with missing information about ASA score (*n* = 23 267) and 1 841 individuals with missing data on cohabitation status, the analytical sample was 165 596 individuals with first-time hip fracture.

### Outcome

The outcome was 1-year mortality registered in the Cause of Death Registry.

### Exposures

The exposure was comorbidity measured in two ways: ASA score and CCI.

ASA score was extracted from the SHR and is estimated by the local anaesthesiologist prior to the hip fracture surgery. In this study, 4 different categories were used: ASA 1, 2, 3 and 4 + 5 (due to a small number of individuals with an ASA score of 5). The calculation of the CCI in this study follows the methods presented by Brusselaers et al. [9]. The diagnoses included in the score are acute myocardial infarction, congestive heart failure, peripheral vascular disease, cerebral vascular disease, dementia, chronic obstructive lung disease, rheumatoid disease, liver disease, diabetes, hemiplegia or paraplegia, renal disease, cancer, metastatic cancer, and AIDS. The corresponding ICD 10-codes used to identify the different diseases in the hip fracture patients and the weighted scores for the respective disease are presented in Supplementary Table 1. In this study, the comorbidities that constitute the CCI were extracted from the NPR by ICD codes from hospitalizations during 1 year prior to the hip fracture and thereafter categorized into 5 different groups: 0, 1, 2, 3, and 4 +.

### Confounders

The data on age, sex, admission date, type of hip fracture (categorized into intracapsular, pertrochanteric and subtrochanteric) and cohabitation status (living alone, cohabiting and institutional housing) before the fracture was retrieved from the SHR. Dementia diagnosis was based on the ICD-codes from the NPR within 1 year prior to the fracture. Education was retrieved from LISA and categorized into three different categories based on the highest attained education (primary, secondary and university).

### Statistics

Descriptive statistics including Kaplan–Meier graphs for survival data were produced. The Kaplan–Meier graphs were calculated for ASA score and CCI, stratified by age and sex. Sex specific survival was analysed with multivariate Cox proportional hazards models controlling for possible confounders. Education level and type of fracture did not alter the results when brought into the model and were hence left out of the analysis. Correlation between ASA score and CCI was calculated with Pearson’s correlation coefficient.

#### Sensitivity analysis

To test the robustness of the results in subgroups of the population, additional survival analyses were carried out, stratifying for age groups and in a subsample of participants with diagnosed dementia.

The statistical calculations were performed with the Stata 15 and 16 (Statacorp, TX).

### Ethics

The SHR is based on consent from participants. The study has been approved by the Regional Research Ethics Board 2011/136-31/5 and complementary approvals 2017/1088-31 and 2018/84-32.

## Results

**Fig. 1:**
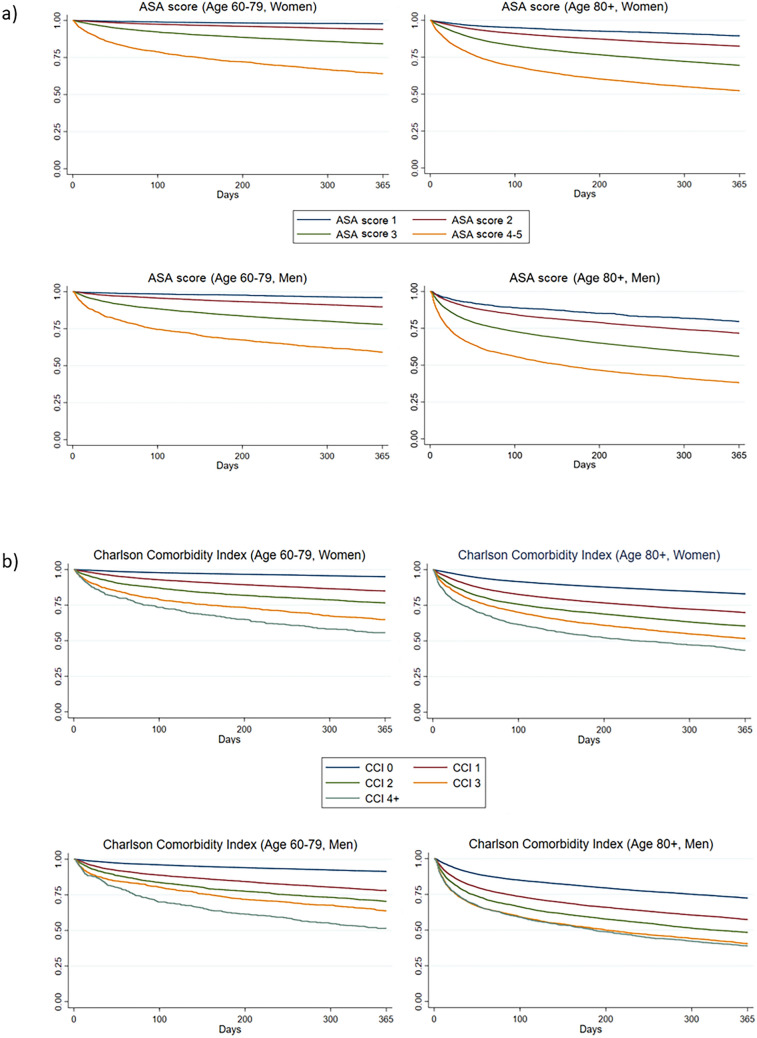
Unadjusted Kaplan–Meier curves for 1-year survival among hip fracture patients according to **a** the ASA score grading and **b** the CCI scoring, stratified by sex and age

The main characteristics of the study population are presented in Table 1. Out of the 165 596 participants, 114 549 (69%) were women. The mean age (± SD) for women at baseline was 82.9 years (± 8.1) and 21.3% had died within one year from the fracture. Among men, the mean age was 81.0 years (± 8.5) and the 1-year mortality was 31.8%. The absolute risks of 1-year mortality according to ASA score and CCI are presented in Table 2. Patients with the lowest ASA (a score of 1) had a 1-year mortality risk of 7.2%, while patients with the lowest CCI (Score 0) had a 1-year mortality risk of 14.7%. The corresponding numbers for highest ASA and CCI categories were 48.8% and 52.2%. The crude Kaplan–Meier survival curves for different ASA- and CCI scores are presented in Fig. 1, stratified for sex and age. The curves show a stepwise increase in mortality per increase in grading for both ASA score and CCI and that the mortality is higher among older individuals and among men, even for low levels of ASA score and CCI. All curves show a steeper slope at the beginning, indicating that the risk of dying is higher within the first couple of months after the fracture.

**Table 1:** Baseline characteristics of the study population presented as absolute numbers (percentage)

	**Women (*n* = 114,549)**	**Men (*n* = 51,047)**
Age, mean (SD)	82.9 (8.1)	81.0 (8.5)
Education level, *n* (%)		
Primary	65,805 (60.9)	26,436 (54.02)
Secondary	31,036 (28.7)	15,791 (32.27)
University	11,137 (10.3)	6714 (13.72)
Cohabitation status, *n* (%)		
Alone	59,997 (52.4)	17,978 (35.2)
Cohabiting	28,114 (24.5)	21,011 (41.2)
Institutional housing	26,438 (23.1)	12,058 (23.6)
Diagnosed dementia, *n* (%)	18,152 (15.9)	7684 (15.1)
ASA score, *n* (%)		
1	8150 (7.1)	3109 (6.1)
2	47,586 (41.5)	16,742 (32.8)
3	50,316 (43.9)	25,414 (49.8)
4–5	8497 (7.4)	5782 (11.3)
CCI, *n* (%)		
0	60,678 (53.0)	21,948 (43.0)
1	34,590 (30.2)	15,378 (30.1)
2	13,519 (11.8)	8410 (16.5)
3	4266 (3.7)	3559 (7.0)
4 +	1496 (1.3)	1752 (3.4)
1-year mortality, *n* (%)	24,423 (21.3)	16,254 (31.8)

**Table 2:** Absolute risk (in %) for 1-year mortality among hip fracture patients according to ASA score and CCI, stratified by sex and age groups

	All	Women		Men	
		60–79	80 +	60–79	80 +
ASA					
1	7.2	2.2	10.5	3.9	20.3
2	15.6	6.1	17.5	10.2	28.3
3	30.2	15.7	30.5	22.2	44.0
4–5	48.8	35.9	47.7	40.9	61.8
CCI					
0	14.7	5.0	17.0	8.6	27.6
1	28.9	15.0	30.1	22.0	42.6
2	39.0	23.4	39.5	29.5	51.7
3	48.5	35.1	48.3	36.2	59.5
4 +	55.2	44.3	56.5	48.5	61.1
Total	24.6	11.0	25.7	17.8	40.5

**Fig. 2:**
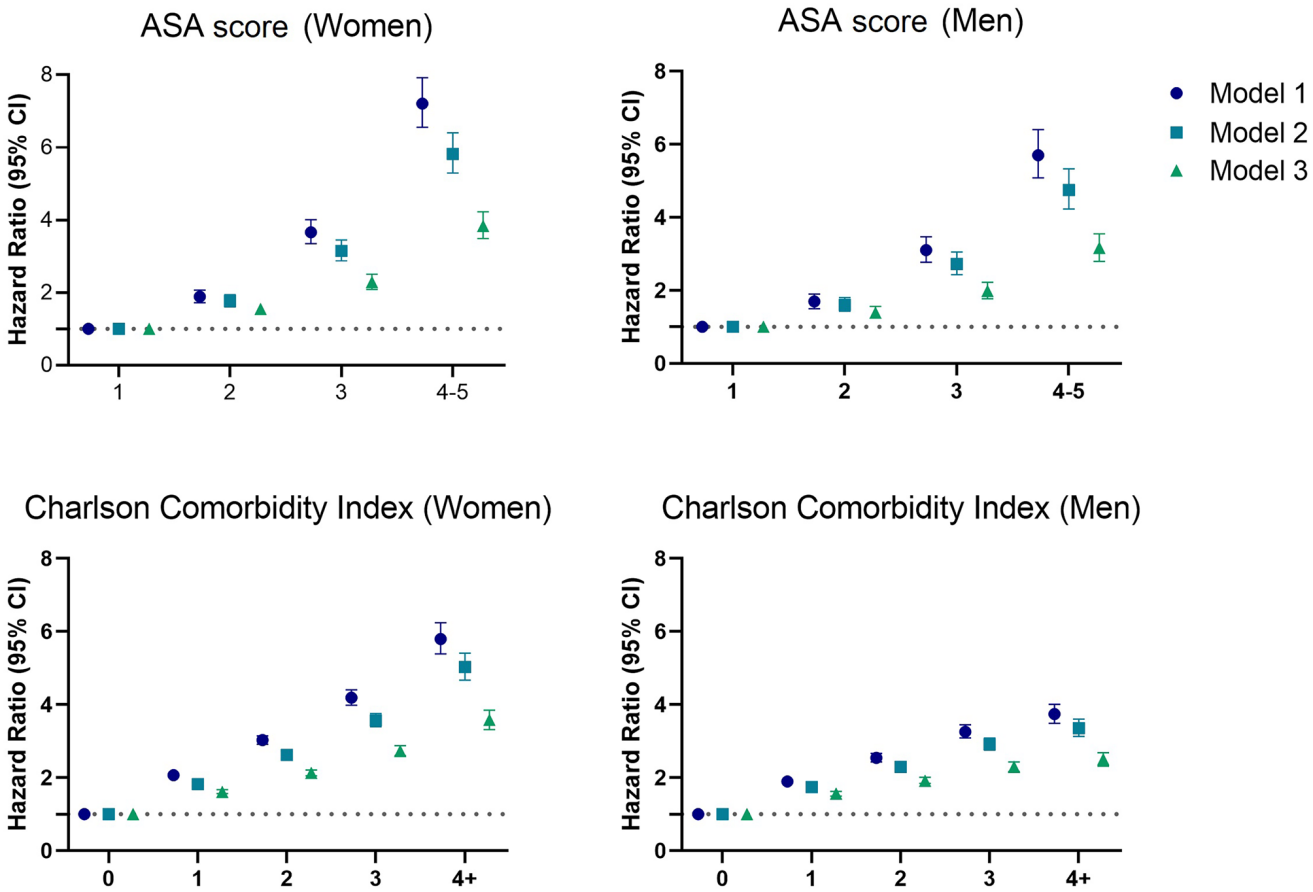
Association between ASA score and CCI with 1-year mortality, analyzed with Cox proportional hazards (HR, 95% significance level). Model 1: controlled for age; Model 2: controlled for age and cohabitation status; Model 3: controlled for age, cohabitation status and the comparing measurement (ASA score or CCI)

The association between ASA score and 1-year mortality, estimated with Cox proportional hazards and adjusted for potential confounders, are presented in Fig. 2 and Supplementary Table 2. The hazard ratios (HRs) for the ASA score showed a stepwise increase in risk for each increase in ASA score which remained in the fully adjusted models, although attenuated. The CCI showed a similar pattern for women, while the HRs for men increased less for each extra point, with a plateauing tendency for the highest scores.

**Fig. 3:**
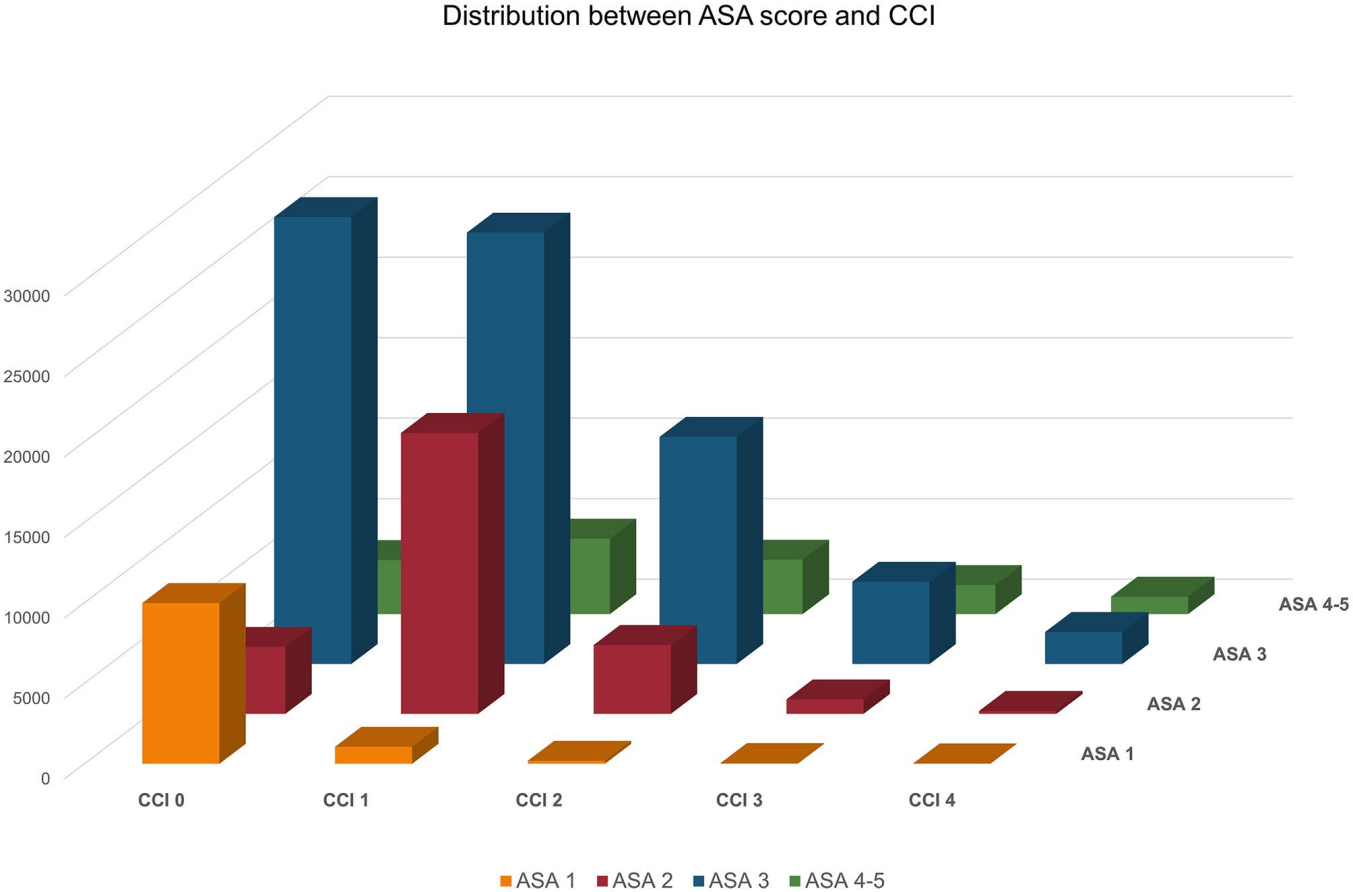
Cross tabulation of the distribution between ASA score and CCI among hip fracture patients

A cross-tabulation of the distribution of the two tools is presented in Fig. 3. Individuals are mainly in similar risk levels regardless of comorbidity being measured by ASA score or CCI, although the two tools seem to detect somewhat different risk profiles, with for example 2% of the individuals being classified as having a CCI 0 while being in the highest ASA category of 4 or 5. The Pearson’s correlation calculations reinforced this finding, with a general correlation of 0.4 for the whole study population, as well as for women and men separately.

### Sensitivity analysis

The survival analyses in different subgroups showed similar patterns as the main analysis, although with an attenuated association among 80 + year old individuals and among individuals with dementia (results not shown).

## Discussion

In this nationwide register study of Swedish hip fracture patients, both ASA score and CCI showed a positive and stepwise association with 1-year mortality among men and women and in both age groups. Although the results that both tools are associated with mortality are in line with the previous studies, [24, 25] our study contributes to the existing knowledge by comparing the two tools in a large nationwide sample of hip fracture patients. We also show that the strengths of the associations between comorbidity and mortality is slightly different between women and men and between age groups. For both women and men mortality increases with increasing ASA score, although the increase is less steep for the younger age group. For women and younger men, the CCI shows similar associations with mortality while we find a plateau around a CCI score of 3 among the older men. This indicates that there is a ceiling effect for how much a heavier comorbidity burden matters when studying the combination of comorbidities that the CCI consists of among older men. This is in line with a Norwegian study showing that already a CCI score of 2 or more was associated with higher mortality, indicating that the highest scores of the CCI might not add much extra information [26]. Although many studies have explored the association between CCI and mortality in hip fracture patients, most have assessed CCI as either continuous or binary (low vs. high) and it is therefore not possible to assess a possible ceiling effect of CCI in these studies. In a recent study, Quach et al. concluded that ASA score was associated with mortality while CCI was not among 320 studied hip fracture patients [21]. In our study, both tools were associated with mortality even after controlling for possible cofounders. This difference might be explained by sample size and type of analysis used but also by selection, since Quach et al.’s study sample was limited to 320 older adults recruited from one orthogeriatric unit.

Our data show that both high ASA score and CCI is more common among men, and men also have a higher 1-year mortality, confirming the results of previous studies [27–29]. However, the two tools’ associations with 1-year mortality are more pronounced in women. This is in line with the previous research from Riska et al., who showed a higher burden of comorbidities and 1-year mortality among men, but a weaker association between comorbidities and mortality compared to women [26]. Kannegaard et al. also studied sex differences in the association between comorbidity and mortality in hip fracture patients. They conclude that the excess comorbidity among men does not account for the increased risk of dying but suggest that the increased mortality for men could be explained by acute complications or undetected chronic conditions that have not been registered prior to the fracture [30]. It could also be interpreted as women being more affected by the type of comorbidities that are captured in these tools, or that unknown factors other than these specific comorbidities impact the outcome after a hip fracture for men. Although sex differences in hip fracture prevalence and mortality among hip fracture patients are well-known, the mechanisms behind this are less studied and need further investigation. When the outcome is regained function and mobility, the results are often reversed, men recover better from a hip fracture than women [31, 32]. This might be explained by selection; more men die after the hip fracture and those who survive are healthier compared with surviving women. In this study, a part of the sex difference might also be explained by the difference in size and mortality rate in the reference groups for women and men, respectively.

One should note the differences in distribution and scales between the two indices, 7% with the lowest ASA score are compared to 50% with the lowest CCI score and for the highest categories the proportions are 9% compared to 2%, respectively. This makes a more in-depth comparison between the tools, such as increase in risk per extra point, difficult. Although, it seems like each extra point in the CCI is associated with an increase in the mortality risk at the lower end of the scale, this risk increase attenuates with higher CCI score (especially among men) in contrast to ASA which showed a more linear pattern of the association with mortality. Even though the HRs for the two scores are rather similar, the correlation analysis reveals that the two tools measure somewhat different aspects of comorbidity. We interpret this finding as the two tools capturing different aspects of health, but that both contain information that is associated with increased mortality risk one year after a hip fracture. This inference is strengthened by the fact that a strong association remains in the final survival analysis models, where the other tool (CCI and ASA score, respectively) is included in the model. If ASA score and CCI were measuring the same thing, the association in the final model had disappeared. Although being strongly associated with 1-year mortality, these tools alone are not enough to capture a complete picture of the risk after a hip fracture. This is also true for more complex tools, such as the O-POSSUM and the NHFS [4], indicating that comorbidity level, although being highly associated with mortality in this patient group, is only a part of a more complex explanation of differences in mortality between individuals. We believe that a measure of comorbidity by itself is not enough to predict mortality in hip fracture patients. As pointed out by Schilling et al., since the ASA score is already existing data in most surgery settings, it might be a cheap and convenient option to evaluate comorbidity, as a part of a prediction model [17].

The CCI were data was gathered from ICD-codes within a year prior to the hip fracture and the ASA score grading was done prior to surgery, normally within days after the fracture. This time aspect might impact the discrepancies between the tools. In an ageing population, the annual change in number of chronic diseases and overall health status can be substantial [33]. In addition, the CCI is developed to measure the burden of specific comorbidity, while the ASA score is developed to optimize perioperative care [10, 13].

The main strengths of this study include a national sample from high-quality registers and followingly a big study sample. Thanks to the linking of the national patient register with a clinical register with more in-depth information, we had the possibility to compare two different measures of comorbidity in the same population. One limitation of this study is that the level of CCI might be underestimated; if a patient indeed had any of the diseases included in the tool but was not treated at a hospital for that disease within a year prior to the hip fracture it would not be present in the CCI variable. In addition, a direct stepwise comparison was not possible due to the different constructs of the two tools. Future studies should aim to move from associations to prediction of mortality among hip fracture patients, by combining important risk factors in prediction models. Additional studies about mechanisms behind the sex differences found in this study and in previous studies are also needed.

In summary, this study showed that both ASA score and CCI are associated with 1-year mortality after a hip fracture in a similar way despite measuring different factors and capturing different individuals at risk. Both tools show a stepwise association with 1-year mortality in hip fracture patients. This is promising since any of the tools can aid in prioritizing vulnerable hip fracture patients at risk of adverse outcomes. Last, even though men have higher mortality after hip fracture, the association between comorbidity and mortality is more pronounced for women with both tools.

## Conclusions and clinical applications

Since both CCI and ASA score show a similarly strong association with mortality in hip fracture patients, despite capturing somewhat different individuals, ASA score may be preferable in a clinical setting because no extra work is needed for health care staff to gather the information.

## Supplementary Information

Below is the link to the electronic supplementary material.

Supplementary file1 (DOCX 25 KB)

## References

[1] Katsoulis M, Benetou V, Karapetyan T et al (2017) Excess mortality after hip fracture in elderly persons from Europe and the USA: the CHANCES project. J Intern Med 281:300–310 10.1111/joim.12586 28093824

[2] Mohamed K, Copeland GP, Boot DA et al (2002) An assessment of the POSSUM system in orthopaedic surgery. J Bone Joint Surg Br 84:735 10.1302/0301-620x.84b5.12626 12188495

[3] Wiles MD, Moran CG, Sahota O et al (2011) Nottingham Hip Fracture Score as a predictor of one year mortality in patients undergoing surgical repair of fractured neck of femur. Br J Anaesth 106:501–504 10.1093/bja/aeq405 21278153

[4] Karres J, Heesakkers NA, Ultee JM et al (2015) Predicting 30-day mortality following hip fracture surgery: evaluation of six risk prediction models. Injury 46:371–377 10.1016/j.injury.2014.11.004 25464983

[5] Pedersen AB, Ehrenstein V, Szepligeti SK et al (2017) Thirty-five-year trends in first-time hospitalization for hip fracture, 1-year mortality, and the prognostic impact of comorbidity: a Danish nationwide cohort study, 1980–2014. Epidemiol 28:898–905 10.1097/EDE.0000000000000729 28767515

[6] Gonzalez-Zabaleta J, Pita-Fernandez S, Seoane-Pillado T et al (2016) Comorbidity as a predictor of mortality and mobility after hip fracture. Geriatr Gerontol Int 16:561–569 10.1111/ggi.12510 25981487

[7] Lunde A, Tell GS, Pedersen AB et al (2019) The role of comorbidity in mortality after hip fracture: a nationwide Norwegian study of 38,126 women with hip fracture matched to a general-population comparison cohort. Am J Epidemiol 188:398–407 10.1093/aje/kwy251 PMC6357811 30407488

[8] Smith T, Pelpola K, Ball M et al (2014) Pre-operative indicators for mortality following hip fracture surgery: a systematic review and meta-analysis. Age Ageing 43:464–471 10.1093/ageing/afu065 24895018

[9] Brusselaers N, Lagergren J (2017) The charlson comorbidity index in registry-based research. Methods Inf Med 56:401–406 10.3414/ME17-01-0051 29582935

[10] Charlson ME, Pompei P, Ales KL et al (1987) A new method of classifying prognostic comorbidity in longitudinal studies: development and validation. J Chronic Dis 40:373–383 10.1016/0021-9681(87)90171-8 3558716

[11] Armitage JN, van der Meulen JH, Royal College of Surgeons Co-morbidity Consensus G (2010) Identifying co-morbidity in surgical patients using administrative data with the Royal College of Surgeons Charlson Score. Br J Surg 97:772–781 10.1002/bjs.6930 20306528

[12] Lau TW, Fang C, Leung F (2016) Assessment of postoperative short-term and long-term mortality risk in Chinese geriatric patients for hip fracture using the Charlson comorbidity score. Hong Kong Med J 22:16–22 10.12809/hkmj154451 26680155

[13] Saklad M (1941) Grading of patients for surgical procedures. Anesthesiol 2:281–284

[14] Dripps RD (1963) New classification of physical status. Anesthesiol 24:111

[15] As a Physical Status Classification System [Internet] (2015) American Society of Anesthesiologists. https://www.asahq.org/standards-and-guidelines/asa-physical-status-classification-system

[16] Soderqvist A, Ekstrom W, Ponzer S et al (2009) Prediction of mortality in elderly patients with hip fractures: a two-year prospective study of 1,944 patients. Gerontol 55:496–504 10.1159/000230587 19628932

[17] Schilling LP, Bozic JK (2016) Development and validation of perioperative risk-adjustment models for hip fracture repair, total hip arthroplasty, and total knee arthroplasty. J Bone Joint Surg 98:e2 10.2106/JBJS.N.01330 26738909

[18] Kastanis G, Topalidou A, Alpantaki K et al (2016) Is the ASA score in geriatric hip fractures a predictive factor for complications and readmission? Scientifica (Cairo) 2016:7096245 10.1155/2016/7096245 PMC4880678 27293978

[19] Donegan JD, Gay NA, Baldwin EK et al (2010) Use of medical comorbidities to predict complications after hip fracture surgery in the elderly. J Bone Joint Surg 92:807–813 10.2106/JBJS.I.00571 20360502

[20] Biz C, Tagliapietra J, Zonta F et al (2020) Predictors of early failure of the cannulated screw system in patients, 65 years and older, with non-displaced femoral neck fractures. Aging Clin Exp Res 32:505–513 10.1007/s40520-019-01394-1 31677126

[21] Quach LH, Jayamaha S, Whitehouse SL et al (2020) Comparison of the Charlson Comorbidity Index with the ASA score for predicting 12-month mortality in acute hip fracture. Injury 51:1004–1010 10.1016/j.injury.2020.02.074 32151423

[22] Meyer AC, Hedström M, Modig K (2020). The Swedish hip fracture register and national patient register were valuable for research on hip fractures: comparison of two registers. J Clin Epidemiol.

[23] Ludvigsson JF, Andersson E, Ekbom A et al (2011) External review and validation of the Swedish national inpatient register. BMC Public Health 11:450 10.1186/1471-2458-11-450 PMC3142234 21658213

[24] Bjorkelund KB, Hommel A, Thorngren KG et al (2009) Factors at admission associated with 4 months outcome in elderly patients with hip fracture. AANA J 77:49–58 19263829

[25] Kirkland LL, Kashiwagi DT, Burton MC et al (2011) The Charlson Comorbidity Index Score as a predictor of 30-day mortality after hip fracture surgery. Am J Med Qual 26:461–467 10.1177/1062860611402188 21450939

[26] Riska BSL, Forsén L, Omsland TK et al (2018) Does the association of comorbidity with 1-year mortality after hip fracture differ according to gender? The Norwegian epidemiologic osteoporosis studies (NOREPOS). J Am Geriatrics Soc 66:553–558 10.1111/jgs.15207 29427505

[27] Sullivan KJ, Husak LE, Altebarmakian M et al (2016) Demographic factors in hip fracture incidence and mortality rates in California, 2000–2011. J Orthop Surg Res 11:4 10.1186/s13018-015-0332-3 PMC4705624 26746904

[28] Haleem S, Lutchman L, Mayahi R et al (2008) Mortality following hip fracture: trends and geographical variations over the last 40 years. Injury 39:1157–1163 10.1016/j.injury.2008.03.022 18653186

[29] Wei J, Zeng L, Li S et al (2019) Relationship between comorbidities and treatment decision-making in elderly hip fracture patients. Aging Clin Exp Res 31:1735–1741 10.1007/s40520-019-01134-5 PMC6825646 30993661

[30] Kannegaard PN, van der Mark S, Eiken P et al (2010) Excess mortality in men compared with women following a hip fracture. National analysis of comedications, comorbidity and survival. Age Ageing 39:203–209 10.1093/ageing/afp221 20075035

[31] Arinzon Z, Shabat S, Peisakh A et al (2009) Gender differences influence the outcome of geriatric rehabilitation following hip fracture. Arch Gerontol Geriatr 50:86–91 10.1016/j.archger.2009.02.004 19303648

[32] Xu BY, Yan S, Low LL et al (2019) Predictors of poor functional outcomes and mortality in patients with hip fracture: a systematic review. BMC Musculoskelet Disord 20:568 10.1186/s12891-019-2950-0 PMC6882152 31775693

[33] Dekhtyar S, Vetrano DL, Marengoni A et al (2019) Association between speed of multimorbidity accumulation in old age and life experiences: a cohort study. Am J Epidemiol 188:1627 10.1093/aje/kwz101 31274148

