# Supplementary material for: Comorbidity and the association with 1-year mortality in hip fracture patients: can the ASA score and the Charlson Comorbidity Index be used interchangeably?
Source: Aging Clin Exp Res. 2021 Jun 9;34(1):129–36. doi: 10.1007/s40520-021-01896-x (PMC8795011; doi:10.1007/s40520-021-01896-x)
Supplement: Supplementary file 1 — Supplementary file1 (DOCX 25 KB) [file 40520_2021_1896_MOESM1_ESM.docx]

# Supplementary material

Supplementary table 1. Calculation of Charlson Comorbidity Index. ICD-codes and points for calculating the CCI.

| Disease | ICD 10 | Score |
| --- | --- | --- |
| Myocardial infarction | I22-I23, I252 | 1 |
| Congestive heart failure | I11, I13, I255, I42–43, I50, I517 | 1 |
| Peripheral vascular disease | I70–73, I770-I771, K551, K558–559, R02, Z958–959 | 1 |
| Cerebrovascular disease | G45–46, I60–69 | 1 |
| Dementia | A810, F00–03, F051, G30–31 | 1 |
| Chronic pulmonary disease | I26–27, J40-J47, J60–67, J684, J701, J703 | 1 |
| Rheumatic disease | M05–06, M09, M120, M315, M32-M36 | 1 |
| Liver disease | B18, I85, I864, I982, K70–71, K721, K729, K76, R162, Z944 | 2 |
| Diabetes mellitus | E10–14 | 2 |
| Hemiplegia/paraplegia | G114, G81–83 | 2 |
| Renal disease | I12–13, N01, N03, N05, N07, N08, N171, N172, N18, N19, N25, Z49, Z940, Z992 | 2 |
| Malignancy | C00–26, C30–34, C37–41, C43, C45–58, C60–76, C80–85, C88, C90–97 | 2 |
| Metastatic tumours | C77–79 | 6 |
| AIDS/HIV | B20–24 | 6 |

Supplementary table 2. Association between ASA score and CCI with 1- year mortality stratified by sex, analyzed with Cox proportional hazards (HR, 95% significance level).

|  |  | | |  | | |
| --- | --- | --- | --- | --- | --- | --- |
|  | **Women** | | | **Men** | | |
|  |  | | |  | | |
|  | Model 1 | Model 2 | Model 3 | Model 1 | Model 2 | Model 3 |
| **ASA score** |  |  |  |  |  |  |
| 1 | ref | Ref | Ref | Ref | Ref | ref |
| 2 | 1.89 (1.72-2.07) | 1.77 (1.61-1.94) | 1.55 (1.41-1.69) | 1.70 (1.5 -1.90) | 1.60 (1.43-1.80) | 1.39 (1.24-1.56) |
| 3 | 3.66 (3.35-4.01) | 3.15 (2.88-3.45) | 2.29 (2.09-2.51) | 3.10 (2.77-3.47) | 2.72 (2.43-3.05) | 1.98 (1.77-2.22) |
| 4-5 | 7.21 (6.56-7.92) | 5.82 (5.30-6.40) | 3.84 (3.49-4.23) | 5.70 (5.08-6.40) | 4.75 (4.23-5.33) | 3.16 (2.80-3.55) |
|  |  |  |  |  |  |  |
|  |  |  |  |  |  |  |
| **CCI** |  |  |  |  |  |  |
| 0 | Ref | Ref | Ref | Ref | Ref | ref |
| 1 | 2.07 (2.01 -2.13) | 1.82 (1.77-1.88) | 1.61 (1.56-1.67) | 1.90 (1.83-1.98) | 1.74 (1.67-1.81) | 1.56 (1.50-1.62) |
| 2 | 3.03 (2.92-3.14) | 2.62 (2.53-2.72) | 2.13 (2.05-2.21) | 2.54 (2.43-2.66) | 2.30 (2.20-2.40) | 1.92 (1.84-2.01) |
| 3 | 4.19 (3.98 -4.40) | 3.57 (3.40- 3.76) | 2.73 (2.60-2.88) | 3.26 (3.09-3.45) | 2.92 (2.76-3.08) | 2.30 (2.17-2.43) |
| 4+ | 5.80 (5.39-6.24) | 5.03 (4.67-5.41) | 3.58 (3.32-3.85) | 3.74 (3.49-4.01) | 3.36 (3.13-3.60) | 2.50 (2.32-2.68) |
|  |  |  |  |  |  |  |

**Model 1**: Controlled for age; **Model 2**: controlled for age and cohabitation status; **Model 3**: controlled for age, cohabitation status and the comparing survival prediction measurement (ASA score or CCI).
